# Physical activity barriers and facilitators among working mothers and fathers

**DOI:** 10.1186/1471-2458-14-657

**Published:** 2014-06-27

**Authors:** Emily L Mailey, Jennifer Huberty, Danae Dinkel, Edward McAuley

**Affiliations:** 1 Department of Kinesiology, Kansas State University, 1A Natatorium, Manhattan, KS 66502, USA; 2 School of Nutrition and Health Promotion, Arizona State University, Phoenix, AZ 85004, USA; 3 School of Health, Physical Education, and Recreation, University of Nebraska Omaha, Omaha, NE 68182, USA; 4 Department of Kinesiology and Community Health, University of Illinois, Urbana, IL 61801, USA

**Keywords:** Physical activity, Parenthood, Working mothers, Fathers, Focus groups

## Abstract

**Background:**

The transition to parenthood is consistently associated with declines in physical activity. In particular, working parents are at risk for inactivity, but research exploring physical activity barriers and facilitators in this population has been scarce. The purpose of this study was to qualitatively examine perceptions of physical activity among working parents.

**Methods:**

Working mothers (n = 13) and fathers (n = 12) were recruited to participate in one of four focus group sessions and discuss physical activity barriers and facilitators. Data were analyzed using immersion/crystallization in NVivo 10.

**Results:**

Major themes for barriers included family responsibilities, guilt, lack of support, scheduling constraints, and work. Major themes for facilitators included being active with children or during children’s activities, being a role model for children, making time/prioritizing, benefits to health and family, and having support available. Several gender differences emerged within each theme, but overall both mothers and fathers reported their priorities had shifted to focus on family after becoming parents, and those who were fitting in physical activity had developed strategies that allowed them to balance their household and occupational responsibilities.

**Conclusions:**

The results of this study suggest working mothers and fathers report similar physical activity barriers and facilitators and would benefit from interventions that teach strategies for overcoming barriers and prioritizing physical activity amidst the demands of parenthood. Future interventions might consider targeting mothers and fathers in tandem to create an optimally supportive environment in the home.

## Background

Declines in physical activity across the transition to parenthood, particularly for mothers, are well-documented [1-4]. Parents face numerous barriers to physical activity (e.g., lack of time, guilt, lack of energy, etc.) and thus exhibit high levels of inactivity as a group [5]. These trends are concerning because inactivity not only has detrimental effects on health and quality of life among parents, but may also impact their children’s behaviors [6]. Thus, developing interventions to promote physical activity among parents is an important public health priority.

Recently, working mothers have been identified as a population that could benefit significantly from interventions to promote physical activity [7]. Over 70% of mothers now work outside of the home, and the combination of work, household and childcare responsibilities leaves little time for personal leisure activities [8,9]. Perceptions of lack of time may also be exacerbated in this population by feelings of guilt associated with taking time away from their children to exercise because the time they have with their children is already limited [10,11]. Furthermore, the “role overload” experienced by many working mothers has been associated with negative health outcomes, including elevated levels of stress, depression, and anxiety [12-14].

One might speculate that working fathers are also increasingly affected by the same “role overload” mothers experience [15,16]. Although fathers have historically played the role of “breadwinner” in families and spent minimal time on household or childcare duties, recent time use data suggests the amount of time married fathers devote to childcare has increased substantially, perhaps because the increase in dual-earner couples demands a greater sharing of family responsibilities [17]. Thus, it is likely that working fathers experience similar barriers to physical activity as their female counterparts, but their perceptions of physical activity have not been examined to date. In particular, the extent to which fathers report that guilt interferes with their physical activity is an interesting question to address. Until recently, guilt has primarily been understood as a barrier unique to females due to deeply rooted cultural discourses about the “ethic of care” (i.e., the notion that a mother’s primary role is to take care of others’ needs before her own) [18,19].

For interventions promoting physical activity among working mothers and fathers to be successful, it is important to have a thorough understanding of physical activity barriers and facilitators within these populations. The extent to which physical activity barriers and facilitators are similar among working mothers and fathers will assist future researchers in designing and tailoring interventions for parents. In particular, it is important to determine whether interventions can target both mothers and fathers simultaneously, or whether unique perceptions of physical activity warrant targeted programs for each gender. Thus, the purpose of this qualitative study was to determine whether parenthood impacts physical activity participation similarly for working mothers and fathers, and to explore barriers to and facilitators of physical activity within these populations.

## Methods

A convenience sample was utilized for this study. Participants were recruited via email lists from two universities in the Midwestern United States to participate in the study. The methods were approved by two Institutional Review Boards (University of Illinois Institutional Review Board #10716; Kansas State University Institutional Review Board #6724) and all treatment of human subjects was conducted in compliance with the ethical standards of the Helsinki Declaration. Individuals who expressed interest in the study completed a brief online screening questionnaire indicating the number and ages of their children, their current employment status, their current exercise habits, and their willingness to participate in an audio recorded 1.5 hour focus group session. To be included in the study, participants had to have at least one child under age 18 living at home and work at least 20 hours per week outside of the home. Participants were not excluded from the study based on their current activity level, but were classified as *active, irregularly active,* or *inactive* according to their self-reported physical activity habits. Active individuals were those who reported engaging in more than 150 minutes per week of moderate/vigorous physical activity. Irregularly active individuals were those who were doing some activity, but not meeting the current physical activity guidelines. Inactive individuals were those who reported no current physical activity.

Eligible participants were scheduled to attend one of four focus group sessions based on their availability. Focus groups were used to promote interaction and simultaneous sharing of a variety of parent perspecives. Data were collected during the summer. Mothers and fathers attended separate sessions and were all from different families. At the beginning of the session, participants signed an informed consent document and completed a brief demographics questionnaire. A trained research assistant led all sessions using a semi-structured interview guide (see Additional file 1). Specific questions were developed to elicit information about physical activity benefits, motives, barriers, and facilitators among parents. In addition, participants were asked to reflect on how their physical activity behavior had changed since becoming a parent. For each topic, the moderator asked follow-up questions to probe for additional information until all conversation had subsided. All sessions were audio recorded.

### Data analysis

Focus groups were transcribed verbatim and uploaded into QSR International’s NVivo 10. Two trained qualitative researchers who were not involved in participation recruitment or data collection and had a combined 16 years of experience analyzed the data through the process of immersion/crystallization [20]. Immersion/crystallization consists of immersing oneself in the data to fully understand the details and then temporarily suspending the process of immersion to reflect on the analysis. Using an inductive process, codes and themes noticed during immersion are identified [21]. One member of the research team read and re-read each transcript to develop initial codes. After initial coding, a second member of the research team read each transcript to refine and add codes. The researchers then met to discuss discrepancies until consensus was achieved. Next, the researchers looked for commonalities between codes to identify overall themes and organized a codebook. The transcripts were then re-coded based on the codebook. Finally, a third member of the research team read the coded data for final confirmation of the overall themes. Nvivo was utilized to facilitate the initial coding and refinement of codes. To further explore the differences between mothers and fathers, once the coding was complete the researchers conducted matrix-coded queries to gain a better understanding of any gender differences. Data were validated through depth of description and exploring alternative interpretations [20]. This study adheres to the RATS guidelines for reporting qualitative research.

## Results

### Participants

A total of 33 mothers completed the initial screening questionnaire. Of these, 19 were scheduled to attend one of the two focus group sessions. Those who were excluded had time/schedule conflicts that prevented them from attending a session (n = 13) or were working less than 20 hours per week (n = 1). Of the 19 participants scheduled to attend a session, six did not attend [family/childcare issues (n = 3), schedule conflict (n = 2), no contact (n = 1)]. Thus, the results presented herein are based on 13 mothers. Demographic characteristics for the subsample of mothers are presented in Table 1.

**Table 1:** Demographic characteristics of participants

**Variable**	**Mean (SD)/Freq (%)**	
	**Mothers (n = 13)**	**Fathers (n = 12)**
Age	38.46 (7.49)	38.83 (4.97)
Number of children	2.08 (0.95)	2.00 (1.21)
Age of youngest child	6.07 (4.49)	3.02 (1.85)
Employment status		
Full-time	13 (100%)	10 (83.3%)
Hours worked per week	44.08 (7.07)	38.96 (8.15)
Marital status		
Married	11 (84.6%)	12 (100%)
Race		
White	12 (92.3%)	10 (83.3%)
African American	1 (7.7%)	2 (16.7%)
Education		
<College graduate	1 (7.7%)	3 (25.0%)
College graduate	4 (30.8%)	2 (16.7%)
Advanced degree	8 (61.5%)	7 (58.3%)
Annual household income		
<$40,000	2 (15.4%)	0
>$40,000	11 (84.6%)	9 (75.0%)
Not disclosed	0	3 (25.0%)
Physical activity level		
Active	6	6
Irregularly active	3	3
Inactive	4	3

Twenty-two fathers completed the initial screening questionnaire. Of these, 15 were scheduled to attend one of the two focus group sessions. Those who were excluded did not respond to a poll to indicate their availability (n = 5) or had other commitments that interfered with the scheduled times (n = 2). Three of the scheduled participants did not attend a session due to schedule conflicts; the remaining 12 participants are included in the analyses. Demographic characteristics for the subsample of fathers are presented in Table 1. Overall, a majority of participants were married, white, highly educated, and working full-time. On average, participants had two children.

### Overview of themes

Mothers’ and fathers’ perceptions about physical activity were clustered around two major categories: barriers and facilitators. Themes related to barriers included family responsibilities (e.g., having children, no childcare), guilt (e.g., family-related, self-related, work-related), lack of support (e.g., spouse, community, role models), scheduling constraints (e.g., lack of time, inconvenient), and work. Themes related to facilitators included participating in activity with children or during children’s activities, being a role model for children, making time or prioritizing (e.g., fitting it into lifestyle, scheduling with spouse, scheduling time), benefits to health and family (e.g., increased energy, reduced stress), and having support (e.g., push from spouse, support from other healthy people). Although the major themes for barriers and facilitators were similar between mothers and fathers, some differences emerged under each theme. Findings are presented below.

### Barriers

#### Family responsibilities

One of the major barriers reported by fathers and mothers was their children. Interestingly, fathers reported their children as a barrier more so than mothers. One inactive father stated, “…Once we had kids my priorities changed a lot. It was like, okay I’m not going out to do this anymore because I gotta find a babysitter, do this, do that. And then once the kids got older it got even more distracting because now we’ve got kids in this activity and this activity at the same time, so you both split off and you don’t get home until 9 o’clock at night. At that point I’m ready to go to bed”. Another said, “…[Exercise] used to be a high priority for me, next to work and my wife, and then I had the child, and it went downhill pretty quickly. Just needs changed, and life took over I guess”. An inactive mother commented, “for me, having kids, I used to walk, and it was nice and everything, and then I had [child], and it all stopped. Completely. It really did”. Mothers also felt that their lack of childcare for exercise time was a barrier. One active mother said, “…that’s the hardest part… somebody that can watch your kids, so they’re not trying to get to you. Like take them to the other room, take them to the park, whatever”.

#### Guilt

Guilt was a barrier for both fathers and mothers. Fathers reported guilt related to family and taking care of themselves whereas mothers reported guilt related to family, taking care of themselves, and work. One inactive father admitted, “…there’s a measure of guilt to it… it’s hard to really justify, well okay, the first thing I’m gonna do is I’m gonna go run, and get way from you guys [family]. You know it’s not what you want to tell them”. Several fathers also reported feeling guilty about taking time away from their wives to exercise. “I feel guilty, like if I’m gonna get up in the morning and go work out, then that might be time where [my wife and I] wouldn’t talk, or we wouldn’t communicate”, explained an irregularly active father. On the other hand, some active fathers said that although they experienced guilt, they did not view it as an impediment: “I think as guys, I don’t know if we’re as motivated by guilt. I think that we probably attack it as a problem that we can solve, and that means, how do we balance it, how do we organize it such that we can do what we want to do, right? As opposed to not do it or do it based on feelings of guilt”. Mothers had similar guilt related to family. An inactive mother explained, “then you feel guilty cause your child’s already been in day care since 7:30 that morning, so you’re gonna extend that… there’s a lot of guilt with that.” An active mother added, “I think when you’re a working mom too, we carry a lot of guilt in some ways, because you’re away, especially when your kids are little”. Mothers in particular worried about taking care of themselves and being judged for participating in physical activity. An active woman shared, “I just had to get over the guilt and the fear that people would judge me by making the choice to do what I needed to do for myself.” Work was another reason mothers felt guilty whereas fathers never mentioned guilt related to work. An active woman admitted, “I usually don’t [extend my lunch hour to exercise] more than twice a week because then I feel guilty about work”. Another active woman stated: “I know people who [block off time to workout during the work day] successfully, but then I think, oh I don’t want people to think I’m that inflexible, or that I’m doing something bad by going to work out at lunch, you know”.

#### Lack of support

Both fathers and mothers reported that not having support was a barrier to their activity. However, fathers more often reported lacking a “community” of others with whom to be active, whereas women mentioned lack of community, lack of spousal support, and not having any role models. One irregularly active father said he would be more active “if I had other guys or something to do it with… or if someone said, hey, come on, going to the gym, then cool, yeah.” Another active father mentioned that he enjoyed sports and so he needed a “community” to play with: “I have to have other people to play with… I don’t do a lot of individual things. I do a lot of competitive things. So I run into the barrier of are there enough people to play with”. Some mothers also indicated parenthood had impacted their opportunities to engage in social physical activities. One active woman stated, “you lack that community… that network to work out with other people”. Only one (inactive) father mentioned that his wife was not supportive: “My wife wanted me to start walking to try and lose some weight and she’d do it with me. That lasted one night. After that I couldn’t get her out of bed… so I lost my drive, it’s like you’re supposed to support me here…” Interestingly, mothers specifically mentioned their husbands’ lack of support being a barrier to their participation in activity. However, this was mostly due to fathers wanting to exercise and not supporting their wives when they wanted to exercise. For example, one active woman said, “I would say, oh I’m gonna work out on these days, and then all of a sudden he’d be like, well I wanna work out… I’d adjust around the schedule that he needed and he’d never go at his time”. Other women mentioned wanting help from role models for support. One active mother said, “It’s really hard to find those really good role models who have balanced everything”.

#### Scheduling constraints

Both mothers and fathers reported scheduling constraints made it difficult to prioritize physical activity after having children, but mothers cited this barrier more often than fathers. Most of the mothers reported having too much to balance in addition to not enough time in the day and thus had a hard time routinely fitting in physical activity. One active woman said, “I wasn’t getting that regular time everyday… one of the boys would get sick…my husband would have to be gone… my routine would totally get wiped out”. Mothers also mentioned that exercise was inconvenient. One inactive woman wouldn’t exercise during her workday because of the inconvenience: “I won’t exercise over my lunch hour because there’s no time to shower and put myself back together”. Fathers similarly reported a lack of time and feeling challenged trying to balance work, children, and exercise: “I find it a challenge to balance the activities… my time, my activities, my family.” Another active father said, “Before [having a family] my whole schedule went around my workouts. So now it’s like okay I have to fit my workout in somewhere, cause I’ve gotta spend time with the kids, and do this, so it’s very different”.

#### Work

Interestingly, work as a barrier to activity was more often mentioned in the focus groups with mothers. One irregularly active mother stated, “I work full-time… I knew I was gonna have to give something up… and exercise was it.” Another inactive mother explained, “We’re just expected to work so many hours. You know, I don’t usually get a lunch hour, let alone a chance to even go use the restroom once, maybe twice a day. And so the thought that I would say, oh sorry guys, you know from 2 to 3:30 I’m gonna go work out… that would be considered I’m not doing my job”. One inactive father described how his type of job was a barrier to activity: “It seems like it’s right when I’ve got everything going that there’s some catastrophe at work that makes me have to put in a 60-hour or 70-hour week, and that derails me cause I’m exhausted. I don’t really feel like…the last thing I want to do is go do anything”. Another active father agreed, “I mean I have stress at work and all that stuff. I don’t want anything extra, you know, I’m just done”.

### Facilitators

#### Being active with children and during children’s activities

One of the major facilitators of physical activity for parents was being able to fit physical activity in with their children or during their children’s activities. One active mother mentioned that she gets her family excited about exercising together: “I’ll say, let’s go exercise guys! Everybody put on your shoes… I’ll do some workouts from one of our tapes and they kind of get into it.” Another irregularly active mother said she exercises during her children’s activities: “I have two very active kids, and my exercise kind of coincides with theirs. The only time I seem to be able to fit anything in is, when they’re at the soccer fields, I walk the soccer fields, or if they’re in the gym, you know, I do the track”. Fathers shared similar stories of incorporating their children and their exercise habits. An active father explained, “I’ve started taking the older one with me to run on Saturday mornings. And I’ll have one that wakes up, you know I do a video workout a few times a week. And I just have them do it right next to me, and they do their thing, and it works out alright”. Another irregularly active father said, “just making him part of the workout, we’ve been able to integrate him more and more and that’s been great. But that’s kind of what brought it back into our lives a little bit, was incorporating him into it. Making him a part of it”.

#### Being a role model for children

Both mothers and fathers also indicated that setting a good example for their children was a valued motive for prioritizing physical activity. Parents expressed that physical activity had taken on added importance in the context of modeling healthy behaviors for their children. One inactive mother said, “My husband and I are both very out of shape, and we don’t want to set that up for our daughter, that, you know, it’s okay to just sit on the couch and watch TV all evening. And that’s where I’m trying to say, it’s nice out, let’s get up and take a walk, and just trying to get it part of the routine, that this is what we do as a family”. Similarly, an active father explained, “The way that I try and view it is, I’ll go to the gym and then I’ll purposely come home with my gym bag, so that the kids see that that’s where I was at. You know, so they can see it’s important to be fit, that type of thing”. Those who were exercising regularly found it very fulfilling when their children expressed an interest in being active as well, even at a very young age. One active mother said, “My 2 ½ year-old, this weekend, he went and put on his shoes and he said, go running mommy? And I think that is really important just for them to be able to put that together, that that’s something important”.

#### Prioritizing

Prioritizing physical activity was also a facilitator that helped mothers and fathers remain active. Both mothers and fathers said that they were able to find ways to fit activity into their lifestyle by carving out time to be active, often while their children were sleeping (early morning or late evening), or during the workday in some cases. Interestingly, more fathers than mothers reported exercising over the lunch hour. One active father stated, “I used to do a lot of my activities after work, but once the baby was born I had to find other times to do it, which is why I do it at lunch time. And I’ve set up my teaching schedule so that I have the noon hour free”. For active mothers, exercising early in the morning was most common, as one mother explained, “I tried the lunch hour thing, I tried right after work. Like I’d leave early and I’d go get the boys after that. I had to just bite the bullet and get up at 5 o’clock in the morning”. Mothers and fathers also reported negotiating with their spouse and “just trying to take over for each other”, or “look at the week ahead of time.” One active mother mentioned she and her spouse have allotted times for exercise: “I run in the morning; he runs after they go to bed”. An active mother also described the value of “…teaching people the skills to negotiate with a partner… like working that into your schedule is really helpful”.

#### Benefits to health and family

Fathers frequently mentioned being motivated to be active because they felt better about their health and their ability to be there for their children. One active father said, “Now that I have a family I’ve gone away from trying to be as big and strong as possible to, I want to be as healthy as possible. I want to be able to be there, and be able to pick them up and go play with them and stuff like that. To have that energy, so that’s my motivation”. Another active father shared, “I find that if I am fit, then all the other parts of my life go a little better. I’m more awake and alert at work, I feel better, I get hurt less, I’m more active playing soccer with my little guy. So that’s one of the reasons I prioritize it, just so everything else in life goes a little smoother”. For active mothers, physical activity was a means of being more present and alert in their roles of parent, spouse, and employee and “just feeling better”. One regularly active mother explained, “Exercise really helps you calm and gives you time to think about things. You know, it really is sort of therapeutic, and it helps you put all [of your other roles] in balance”. Another active mother discussed how being active allows her to relieve stress: “I think it helps me with the kids, you know, I think I’m a much more even-tempered person when I have been exercising, and you know if they do something, then I’m not apt to like, get really angry”.

#### Support

Fathers reported that support in general was important to helping them be active and some mentioned that having a “push” from their wife or support from their wife helped them to be active. One irregularly active father said, “she has really helped me… she’s pushed me to where I am. I do feel a support, like if I wanted to go run, ever, I just feel like she would pick up the slack at home”. Another active father shared something similar: “So yeah, we push each other, as far as working out. I enjoy that”. One regularly active father said, “My wife’s very supportive but it’s because, you know, from the very beginning I told her, I said this is an important part of who I am. But I also try and give back, give her time”. Mothers reported that support from other healthy people motivated them to be active. An active mother explained, “I have to make choices about who I surround myself with on a regular basis… I have to make conscious decisions about, I’m gonna go with this group cause this is a much healthier choice for me today”. One irregularly active mother used her healthy friends as a challenge to herself: “A huge number of my friends… are non-working moms… they have these great figures. Their kids are in school, and they’re working out, and oh, I went for a run today, and I did this, or yoga class was great… to me they’re my challenge, to try and be in as good of shape as they are”.

## Discussion

The purpose of this study was to explore physical activity barriers and facilitators among working mothers and fathers. Overall, working mothers and fathers had similar perceptions of physical activity. Parents expressed that their priorities had shifted after having children, and unanimously conveyed the sentiment that it is no longer “all about me” once you have children. Both active and inactive mothers and fathers reported prioritizing family over exercise, and those who were exercising regularly made it clear that they were not exercising at the expense of time with their children. Instead, they were carving out time to be active during the early morning, lunch hour, or late evening, or doing activities that incorporated their children. Active parents were also adept at seeking support from others, and particularly their spouses, in order to make time for physical activity amidst other occupational and household demands.

Because parents viewed their families as a priority, many reported feeling guilty for taking time away from their families to be active. Such feelings are commonly reported among mothers in particular [22], and may be magnified among working parents whose time with their children is already limited [10,11]. To date, the extent to which fathers also report guilt as a barrier to physical activity has not been explored. The results of this study suggest that fathers experience guilt to a similar degree as mothers, but may be less likely to report that guilt prevents them from being physically active. Several fathers also reported guilt related to taking time away from their wives, because they felt like they needed to prioritize time for their marital relationship as well.

Both mothers and fathers who were consistently making time for physical activity in their lives focused primarily on benefits that were relevant to their role as parents. They wanted to “feel good” and have the energy to enjoy time with their children. Active mothers, in particular, viewed physical activity as a means to de-stress and have an outlet from all of their other demands. These findings are consistent with previous research that has demonstrated intrinsic motives related to daily well-being are associated with improved long-term physical activity maintenance [23]. In essence, active parents were able to alleviate feelings of guilt by viewing physical activity as something that enhanced, rather than detracted from, their ability to be good parents [19]. This led them to autonomously endorse and pursue an active lifestyle because they valued the benefits. Consistent with this notion, mothers and fathers also indicated that setting a good example for their children was a valued motive for prioritizing physical activity. Parents expressed that physical activity had taken on added importance in the context of modeling healthy behaviors for their children, so it did not feel like a selfish activity. Many parents also discussed incorporating their children in their workouts as a way to enjoy an active lifestyle and spend time together. Future interventions might consider providing parents with a variety of age-appropriate ideas for being active with their children, which would not only reduce barriers related to guilt and childcare constraints, but could also have a positive impact on their children’s physical activity participation. Alternatively, if parents want physical activity to serve as an “outlet” or a break from their parenting duties, interventions can still work to alleviate guilt by linking physical activity outcomes with parents’ core values (e.g., reduced stress helps them to be more patient parents) [22]. Such strategies are consistent with empirically supported self-determination theory approaches, which aim to promote sustained behavior change by providing an autonomy-supportive environment to foster the development of internalized motives [24,25].

Although both active and inactive mothers and fathers reported numerous physical activity barriers, those who were engaging in regular physical activity had made a conscious decision to be active because they valued the benefits, and were using a variety of strategies to prioritize active behaviors. Effective strategies included negotiating with a spouse, waking up early to exercise, rearranging work schedules to fit in physical activity, and planning to be active during children’s activities. These strategies epitomize self-regulation, a construct that is consistently incorporated in social cognitive theory-based interventions. Bandura [26] contends that self-regulation (i.e., guiding one’s own actions by setting personal goals and planning courses of action to achieve them) is essential for maintaining a complex behavior such as physical activity. Individuals’ positive perceptions of the behavior provide the motivation to prioritize it, and self-regulatory strategies are instrumental in translating their intentions into actions [27]. Several recent interventions have shown planning/scheduling is a key predictor of physical activity maintenance over time [7,28]. For working parents whose schedules are overloaded and discretionary time is limited, maintaining an active lifestyle is likely to necessitate advance planning to make time for physical activity. Interventions should teach participants self-regulatory skills such as goal setting, action planning, and coping planning to facilitate the behavior change process; there is strong evidence that incorporating such strategies will enhance intervention effectiveness [29,30].

The results of this study also suggest receiving support from others is instrumental in promoting physical activity among working parents. Mothers and fathers discussed the importance of surrounding themselves with active friends and role models, but most often they expressed a need for support from their spouse. These findings add to previous research that has identified spousal support as a key facilitator of physical activity among parents [1,8,31]. Most regularly active mothers and fathers in this study reported that their spouse was also active, and because physical activity was mutually valued the couple negotiated with one another to ensure both parties could carve out time to exercise. These results suggest future interventions should teach parents to seek support from their spouse and to practice negotiating and planning skills so that each individual can prioritize his/her preferred leisure activities. Social support is most effective when it matches the needs of the recipient [32], and working parents are likely to benefit from instrumental support in particular, through which partners provide tangible aid in the form of assistance with household and childcare duties [33]. Several studies have shown the effectiveness of physical activity interventions is enhanced when partners participate together; thus, interventions might also consider targeting mothers and fathers in tandem to create an optimally supportive environment in the home [34,35]. It should be noted, however, that parents may face additional barriers related to seeking social support, including feeling guilty for asking for help or facing logistical challenges associated with providing reciprocal support [33]. Additional research is needed to elucidate optimal sources of social support for parents and effective strategies for targeting these sources in interventions.

One notable difference between males and females was the extent to which they believed they could take time out of the workday to be active. Several men discussed arranging their work schedules in such a way that they could take time to exercise over their lunch hour. Women, on the other hand, perceived additional barriers related to this strategy. Several women expressed concerns that others would perceive them to be less committed to their jobs if they left during the day to exercise, and thus felt guilty for taking time to be active during the workday. Indeed, these concerns are warranted as previous research has demonstrated parents are perceived as less committed and less available to their jobs than non-parents. This is particularly true for working mothers, who are held to strict standards (by themselves and others) in order to demonstrate that caretaking responsibilities are not impinging on occupational duties [36]. These findings underscore a need for interventions designed to facilitate more supportive work environments in which the health and wellness of employees are valued and prioritized. Emerging evidence documenting a significant return on investment for employers who invest in wellness programs provides a compelling rationale for workplaces to take steps to change social norms and promote a “culture of wellness” [37]. Although this area of intervention research is still in its infancy, most public health experts agree that worksite wellness programs will be more likely to produce sustained behavior changes when they incorporate environmental, policy, and programmatic changes to facilitate a shift in workplace norms [38]. Such changes could have significant effects on the physical activity opportunities and perceptions of working parents.

This study has several limitations. Most notably, the sample was relatively small and homogeneous. In particular, participants were highly educated/affluent, so these findings should not be generalized to parents of a lower socioeconomic status who might report different physical activity barriers and facilitators. The present sample had adequate resources to be physically active so the results must be interpreted in that context. Furthermore, almost all participants were married, so the results do not reflect the perceptions of single parents. Despite these limitations, this study makes an important contribution by shedding light on the physical activity perceptions of an inactive but understudied population. In this sample, the barriers and facilitators parents reported were unrelated to their children’s ages, suggesting that these factors are relevant across all stages of parenthood.

## Conclusions

The results of this study suggest working mothers and fathers face similar barriers to being physically active. Efforts to increase physical activity within these populations should draw on prominent behavioral theories (e.g., Self Determination Theory, Social Cognitive Theory) that emphasize tapping into autonomous motives and teaching self-regulatory strategies to increase individuals’ confidence to adopt and sustain an active lifestyle. Specifically, interventions might focus on highlighting physical activity benefits that are relevant to the whole family, teaching creative and convenient ways to prioritize physical activity, and providing a supportive environment, both in the workplace within the home. Working parents prioritize their families first and foremost, but can reduce the perceived dichotomy between self and family by embracing benefits that are consistent with their notions of what it means to be a good parent, incorporating their children in their activities or planning to be active when it will not interfere with time with family.

## Competing interests

The authors declare that they have no competing interests.

## Authors’ contributions

ELM conceptualized and designed the study, conducted the focus groups, transcribed the data, and drafted the manuscript. JH analyzed and interpreted the data and assisted with manuscript preparation. DD analyzed and interpreted the data and assisted with manuscript preparation. EM assisted with study conceptualization and manuscript revision. All authors read and approved the final manuscript.

## Pre-publication history

The pre-publication history for this paper can be accessed here:

http://www.biomedcentral.com/1471-2458/14/657/prepub

## Supplementary Material

Additional file 1 Core questions used to guide focus groups. Click here for file
