# Supplementary material for: Physical activity barriers and facilitators among working mothers and fathers
Source: BMC Public Health. 2014 Jun 27;14:657. doi: 10.1186/1471-2458-14-657 (PMC4227023; doi:10.1186/1471-2458-14-657)
Supplement: Additional file 1 — Core questions used to guide focus groups. [file 1471-2458-14-657-S1.docx]

**Additional File 1**. Core questions used to guide focus groups

1. What are the greatest benefits of exercise that you personally experience?

 2. At this point in your life, is exercising regularly an important goal to you? Why or why not?

 3. What keeps you from exercising as much as you’d like to? What would you consider to be your biggest barriers?

4. How did your physical activity behavior change when you became a parent, if at all? As a parent, have certain periods of time been more difficult than others?

5. What types of help/support do you get (or would you like to get) from other people that allows you to make time for exercise?
